# Juvenile Dermatomyositis Magnetic Resonance Imaging Score (JIS) does not correlate with criteria for clinically inactive disease: a single-centre retrospective evaluation

**DOI:** 10.1007/s00296-021-05049-1

**Published:** 2021-11-18

**Authors:** Kapil Gargh, Eslam Al-Abadi, Samantha Low, Kathryn Harrison, William Coles, Penny Davis, Karl Johnson

**Affiliations:** 1 Childhood Arthritis and Rheumatic Diseases Unit, Birmingham Women’s and Children’s Hospital NHS Foundation Trust, Steelhouse Lane, Birmingham, B4 6NH West Midlands UK; 2 Radiology Department, Birmingham Women’s and Children’s Hospital NHS Foundation Trust, Birmingham, West Midlands UK; 3 Institute of Applied Health Research, College of Medical and Dental Sciences, University of Birmingham, Birmingham, West Midlands UK

**Keywords:** Biomarker, Juvenile dermatomyositis, Magnetic resonance imaging, Paediatric rheumatology

## Abstract

The Paediatric Rheumatology International Trials Organisation (PRINTO) criteria for clinically inactive disease (CID) and their proposal for glucocorticoid tapering do not consider MRI findings, despite the growing use of MRI and development of reliable MRI scoring tools. We aim to evaluate how CID correlates with MRI scores and physician decision making. We retrospectively used the Juvenile Dermatomyositis Imaging Score (JIS) to score MRIs of all children with JDM over a 10-year period. Demographic, diagnosis, treatment and core set measures data were collected. Correlation between CID and JIS was assessed as well as correlation with the physician treatment decision. There were 25 patients with 59 follow-up episodes to analyse correlation between physician treatment decision and JIS; and 50 episodes for the CID category and JIS correlation. JIS was not significantly associated with the CID category but did correlate with the physician decision. No significant association was found between clinical decision and CID category. The JIS area under the ROC curve (AUC) was 0.80 (95% CI 0.62–0.99) with a score ≥ 8 to predict an escalation. JIS sensitivity and specificity were both 78% with accuracy of 78%, compared to only 67%, 46% and 49%, respectively, for the CID criteria. Clinical criteria alone are not sufficient to assess disease activity status. Clinical decision trends correlated to MRI findings but not PRINTO CID criteria. Multi centre prospective studies are needed to replicate our findings and establish how to best use MRI as a biomarker of disease activity.

**Supplementary Information:**

The online version contains supplementary material available at 10.1007/s00296-021-05049-1.

## Introduction

Juvenile dermatomyositis (JDM) is the commonest childhood inflammatory myopathy [1]. Glucocorticoids significantly reduced mortality [2], however; significant morbidity persists, with severe early disease course a risk for poor outcomes [3]. Accurate assessment of disease activity is essential to enable timely escalation/weaning of therapy, achieve disease control and ultimately remission while minimising treatment toxicity [4].

To enable these goals, the Paediatric Rheumatology International Trials Organisation (PRINTO) trials provided the evidence for using methotrexate as a steroid- sparing agent in children with JDM [5], derived criteria for clinically inactive disease (CID) and proposed a regimen of glucocorticoid tapering/discontinuation [6, 7]. The CID require 3 out 4 criteria to be met to define clinically inactive disease: Creatinine Kinase (CK) ≤ 150, Manual Muscle Testing 8 (MMT8) ≥ 78/80, Childhood Myositis Assessment Scale (CMAS) ≥ 48/52 and Physician Global (PG) ≤ 20/100. However, after the acute phase, muscle enzymes are not a reliable marker of muscle inflammation [8]. In addition, clinical examination of muscle power and function are not always reflective of disease activity and may represent a child’s inability to understand and/or perform the tests [9, 10], muscle damage [3], steroid toxicity or non-adherence to rehabilitation efforts. These measures have a ceiling effect and are subject to variation influenced by the assessors training, experience and interpretation of findings [10]. Therefore, the lack of a reliable and reproducible biomarker of disease activity has the potential to prolong the use of steroids in some children while prematurely weaning in some who will subsequently flare.

MRI is already the most commonly used investigation to aid the diagnosis of JDM and the most likely to show an abnormality [11]. A European consensus guideline supported the use MRI to monitor disease activity [12]. The recently developed and validated Juvenile dermatomyositis Magnetic resonance imaging Score (JIS) was designed for quantifying muscle inflammation, with good inter and intra observer reliability and has the potential to be a useful additional tool in assessing disease activity [13, 14]. In our tertiary paediatric rheumatology centre we have regularly used MRI as an adjunct to clinical findings to aid the diagnosis of remission and hence treatment escalation or tapering. We reviewed our practice to evaluate how, in children with JDM attending follow-up appointments, MRI correlates with the PRINTO CID and steroid adjustment decisions taken by the physician.

### Objectives

Our study’s primary objective is to assess if the PRINTO criteria for CID correlate with MRI findings at follow-up. The secondary objective is to assess if the physician’s treatment decision making in relation to weaning or escalating steroids better correlated with: (a) PRINTO criteria for CID or (b) MRI score at the time of the follow-up.

## Methods

### Study design

We conducted a retrospective case notes review of children diagnosed with JDM and retrospectively scored their MRIs using the Juvenile Dermatomyositis Magnetic Resonance Imaging Score (JIS).

### Patients

All patients with a diagnosis of JDM at our tertiary paediatric rheumatology centre between 1st January 2008 and 31st December 2018 were initially included. Clinical information was gathered from case notes, departmental database and hospital clinical systems. Patients were excluded if (i) the data was incomplete, (ii) did not have a follow-up MRI scan, (iii) the eventual diagnosis was not JDM, (iv) the follow-up visit was after the first 2 years of diagnosis (v) there was an interval of > 2 weeks between MRI and clinical assessment or (vi) a change in management was actioned before the MRI was performed.

### Data collection

Data was collected from the baseline visit and each follow-up visit when an MRI was performed: demographics, clinical characteristics, and components of the PRINTO criteria for CID as well as JIS at diagnosis and follow-up. Escalation of treatment was recorded when there was an increase in the dose of steroids, while weaning was recorded if steroids were reduced. Muscle function was assessed by the Childhood Myositis Activity Score (CMAS) with a score between 0 and 52. Muscle strength was assessed by Manual Muscle Testing of 8 muscle groups (MMT8) with a score between 0 and 80.

### Measurement

The MRI results were scored by an experienced paediatric musculoskeletal radiologist using the JIS.

### Procedures

MRI of pelvis and thighs were performed on the same day as clinical assessment using a 1.5 Tesla Siemens scanner. The protocol images were T1 and STIR sequence in axial plane and T2 fat saturated sequence in coronal plane, without contrast.

### Statistical analysis

Association between JIS and each of the CID components was examined using Spearman’s rank correlation. Mann–Whitney test was used to compare JIS and CID outcome in each patient. Association between CID and clinicians decision was examined using Fisher’s exact test. The Kruskal–Wallis test was used to examine the correlation between JIS and the clinician’s decision. The ROC curve was used to assess the association between the different variables and to choose an appropriate cut-point for the MRI score for the best prediction of clinician decision. Final analyses examined both the categorised JIS and CID measure in the prediction of the clinician decision to escalate treatment. Diagnostic performance was calculated by the sensitivity, specificity, positive and negative predictive values and overall accuracy. Corresponding confidence intervals for all statistics were calculated using the exact binomial method. Subsequently a set of sensitivity analyses were performed, when only the first follow-up measurement for each patient was included in the analysis. All data was examined using Stata (version 15.1).

### Ethical approval

This study was registered with the Institution’s Audit office and in accordance with the UK National Research Ethics Service guidance, neither individual informed consent nor formal research ethics committee review was required as the study was undertaken by the direct clinical care team using information previously collected in the course of routine care.

## Results

### Patients and demographics

Between 1st January 2008 and 31st December 2018, twenty-five patients fulfilled the inclusion and exclusion criteria (Supplementary Figure S1). Patient characteristics are shown (Supplementary Table S1).There were 59 individual measurements from distinct follow-up episodes, where an MRI was performed.

### Assessing the associations between the JIS and individual components of the CID

Only the CMAS and physician global score showed true association with the JIS. However, the association was weak (Supplementary Table S2 and Supplementary Fig. S2A and B).

### Assessing the association between JIS, CID criteria clinical decision

**Fig. 1:**
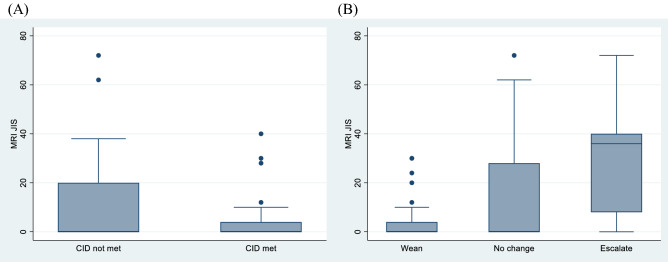
Boxplot association between JIS and overall CID categorisation / clinician decision. Boxplot **A** shows the association between JIS in patients when CID criteria were met and when not met. Boxplot **B** shows the association between JIS and the clinician treatment decision. *CID* clinically inactive disease, *JIS* Juvenile Dermatomyositis Magnetic Resonance Imaging Score

There was sufficient clinical data to determine CID category in 47 clinical episodes (Table 1). The results suggested that JIS was not significantly associated with the CID category. However, the JIS was significantly associated with the clinical decision (Table 1 and Fig. 1A and B). The scores were highest in the escalation group and lowest in the group, where treatment was weaned. On the other hand, there was no significant association was found between clinical decision and CID category (Table 2).

**Table 1:** Association between JIS and overall CID categorisation / clinician decision

Variable	Category	*n*	JIS median [IQR]	*P* value
CID criteria	Not met	21	0 [0, 20]	0.18
	Met	26	0 [0, 4]	
Clinician decision	Wean	35	0 [0, 4]	**0.003**
	No change	15	0 [0, 28]	
	Escalate	9	36 [8, 40]	

Results that reached statistical significance is bold

The first set of figures show the number of measurements in each category either as met or not. The second set of figures presents the median MRI score in each category, along with a corresponding inter-quartile range. These summary measures were used due to the skewed distribution of the MRI scores. *P* values indicating the significance of the association between JIS and the clinical decision but not JIS and the CID criteria

*CID* clinically inactive disease, *JIS* Juvenile Dermatomyositis Magnetic Resonance Imaging Score

**Table 2:** Association between overall CID categorisation and clinician decision

Clinician decision	CID not met, *n* (%)	CID met, *n* (%)	*P* value
Wean	10 (48%)	18 (69%)	0.14
No change	9 (43%)	4 (15%)	
Escalate	2 (10%)	4 (15%)	

The figures are the number and percentage of patients in each clinician decision group for patients, where the CID criteria were and were not met. The results show no correlation between the clinicians decision and the CID criteria

*CID* clinically inactive disease

### ROC analysis

The association between the JIS and the clinician decision was investigated further, with the use of ROC curves to help choose an appropriate cut-point. The fitted ROC curve is shown graphically (Supplementary Fig. S3). The area under the ROC curve (AUC) was 0.80 (95% CI 0.62–0.99). This is a relatively high value, suggesting some diagnostic ability of this measure to predict the clinician decision. The ROC curve analysis was also used to determine the optimal cut-point for predictive purposes. This was chosen as the point which optimised the combination of sensitivity and specificity. The analysis suggested a score ≥ 8 would predict an escalation. Of the 59 observations, the score met this cut-off in 18 (31%) of instances. A full list of the results using all possible cut-offs is shown (Supplementary Table S3).

### Diagnostic performance of JIS and CID in predicting the clinical decision

The analyses suggested that the MRI score had a superior diagnostic performance for the prediction of the clinician decision to escalate. JIS sensitivity and specificity were both 78% with an overall accuracy of 78%, compared to only 49% for the CID criteria. There was a relatively low PPV, but the NPV was high at 95% (Table 3).

**Table 3:** Performance of JIS and CID for the prediction of the clinician decision

Statistic	JIS estimate (95% CI)	CID estimate (95% CI)
Observations in analysis	59	47
Sensitivity	78% (40%, 97%)	67% (22%, 96%)
Specificity	78% (64%, 89%)	46% (31%, 63%)
Positive predictive value	39% (17%, 64%)	15% (4%, 35%)
Negative predictive value	95% (84%, 99%)	91% (70%, 99%)
Accuracy	78% (65%, 88%)	49% (34%, 64%)
Positive likelihood ratio	3.5 (1.9, 6.6)	1.2 (0.7, 2.3)
Negative likelihood ratio	0.29 (0.08, 0.98)	0.72 (0.22, 2.34)

The calculated values are shown, along with corresponding confidence intervals. JIS is more sensitive and specific with better accuracy when compared to CID

*CID* clinically inactive disease, *JIS* Juvenile Dermatomyositis Magnetic Resonance Imaging Score

## Discussion

The PRINTO trials have demonstrated the difficulty in weaning steroids in JDM and the need for reliable biomarkers of disease activity to inform clinical decision making [5, 7, 15]. Our study demonstrated that there does not always appear to be an association between criteria for CID and JIS or physician treatment decision, suggesting that these criteria alone are not sufficient to assess disease activity and accurately inform treatment decisions. On the other hand, there was an association between clinical decision making and JIS. Interestingly, on analysis at the time of first follow-up MRI, the clinical decision-making trends were associated with both CID and JIS, although better with JIS. Finally, the physician global (PG) of diseases activity correlated with the JIS in later stages while at first follow-up CMAS correlated better. The results suggest that the criteria for CID are sensitive to change and informative to the physician early on in the disease course, while MRI was informative in both early and later disease courses, suggesting that its sensitivity is maintained over time.

Current criteria for inactive disease do not use MRI in defining disease inactivity, in steroid tapering or treatment decision making [6, 7, 12]. They are extremely valuable when there is no access to MRI and when there is no clinical doubt. However, MRI is a sensitive method of detection and localisation of muscle inflammation in JDM compared to CK levels [16]. It is useful in assessing equivocal or subclinical disease, and differentiates ongoing inflammation from the effects of longstanding disease on muscle [17, 18].

There is a need to establish if MRI should be used as part of the definition of disease inactivity. When to, and who should, have an MRI? Studies found that MRI can show evidence of inflammation during follow-up or times of clinical doubt, can help asses disease activity and was useful when change in treatment was planned or a flare was suspected[19, 20]. When treatment response plateaued, MRI can compare the degree of inflammation longitudinally and identify patients wrongly assessed as being in remission by clinical criteria only [18]. A study demonstrated that with equivocal clinical and lab parameters, the clinician’s decision to treat was based on the MRI findings and the MRI was also informative when there was no evidence of flare as unnecessary treatment was avoided in 70% of patients [19].

To our knowledge, this is the first study to compare a MRI score of inflammation burden to (i) criteria of disease inactivity and (ii) physician treatment decision making. Our study reports a higher number of follow-up episodes. All MRIs were scored by the same radiologist, limiting inter-observer variability. New strategies are required to assess disease activity accurately; JIS could be a valuable tool in making such an assessment, since it provides objective and quantitative measurement of disease activity, making it a reliable biomarker of disease severity and response to therapeutic interventions in children with JDM [14].

The study is limited by its retrospective single centre nature and small sample despite having the largest number of follow-up MRI’s in the literature. We cannot be certain of the clinician’s intention to treat before the MRI report even though the JIS was not available then. We recognise this bias and, therefore, recommend that a multicentre prospective study is required to validate our observations. MRI may help confirm a diagnosis of an amyopathic disease or amyopathic flare events; however, our findings would not otherwise affect the assessment of disease activity in this subgroup.

In conclusion, we found no correlation between the criteria for CID and muscle inflammation on MRI. Overall, clinical decision trends correlated to MRI findings but not CID. This suggests clinical criteria alone are not sufficient to assess disease activity status. MRI findings were informative to the physician decision making throughout the disease course. Where MRI is available, we recommend it is performed to aid defining disease inactivity and informing the physician’s treatment decisions. Future studies should prospectively assess the correlation between MRI findings and criteria for CID as well as the influence of MRI findings on the physician’s treatment intentions.

## Supplementary Information

Below is the link to the electronic supplementary material.

Supplementary file1 (DOCX 76 KB)

## Data Availability

Data are available from the author upon reasonable request.

## Abbreviations

AUC: Area under the ROC Curve
CID: Clinically inactive disease
CK: Creatinine kinase
CMAS: Childhood Myositis Assessment Scale
JIS: Juvenile Dermatomyositis Magnetic Resonance Imaging Score
MMT8: Manual Muscle Testing 8
MRI: Magnetic resonance imaging
NPV: Negative predictive value
PG: Physician global
PRINTO: Paediatric Rheumatology International Trials Organisation
PPV: Positive predictive value

## Code availability

All data was examined using Stata (version 15.1).
