# Supplementary material for: Juvenile Dermatomyositis Magnetic Resonance Imaging Score (JIS) does not correlate with criteria for clinically inactive disease: a single-centre retrospective evaluation
Source: Rheumatol Int. 2021 Nov 18;42(7):1221–6. doi: 10.1007/s00296-021-05049-1 (PMC9203389; doi:10.1007/s00296-021-05049-1)
Supplement: Supplementary file 1 — Supplementary file1 (DOCX 76 KB) [file 296_2021_5049_MOESM1_ESM.docx]

**Supplementary Information**

**Statistical Methods**

The analyses focussed on the association between three measures; a clinical criterion based on four individual components, the MRI JIS score and the physician’s decision on whether and how to change treatment.

The association between the clinical criteria and MRI score was examined first. Initially the association between the MRI score and each clinical component was examined. All variables were measured on a continuous scale. As the MRI scores were found to have a heavily positively skewed distribution, Spearman’s rank correlation was used for these analyses. The clinical scores were used to calculate whether the CID criteria were met or not (yes/no). The Mann-Whitney test was used to compare the MRI scores between patients where these criteria were or not met.

The association between clinical criteria and the clinician decision was examined. As both variables were categorical in nature, Fisher’s exact test was used to examine the association between the measures. This was preferred to the Chi-square test due to the relatively small numbers in some categories.

The Kruskal-Wallis test was used to compare MRI scores between the three clinician categories, again used due to skewed distribution of the MRI scores. The association between these variables was further examined via the use ROC curves. For this analysis, the clinician decision was categorised as an escalation or not. The ROC curve was also used to choose an appropriate cut-point for the MRI score for the best prediction of clinician decision. The optimal cut-point was chosen as the point which maximised the combination of specificity and sensitivity.

The final analyses examined both the categorised MRI score and CID measure in the prediction of the clinician decision to escalate treatment. Diagnostic performance was calculated by the sensitivity, specificity, positive and negative predictive values and overall accuracy. Corresponding confidence intervals for all statistics were calculated using the exact binomial method.

Initially all the analyses were used using all available data. Subsequently a set of sensitivity analyses were performed, when only the first follow-up measurement for each patient was included in the analysis.

**Sensitivity analysis results**

All analyses were repeated using only the first measurement per patient and resulted in 2 significant differences. First, only the CMAS, and not the physician global, was significantly associated with the JIS. Second, the CID category was significantly associated with the clinical decision. However, JIS ROC curve was higher at 0.95 and still had a higher predictive ability compared to the CID (68% vs 49%) (Supplementary materials Tables S4-7 and Figures S4A&B).

5 Met exclusion criteria:

4 MCTD

1 No FU MRI

59 Follow up episodes for

MRI / physician treatment decision analysis

50 Follow up episodes for:

(i) PRINTO/MRI analysis

(ii) PRINTO/ Physician treatment decision analysis

25 patients included in the final analysis

Met Inclusion Criteria

28

Total number of patients on the DAWN database with diagnosis of JDM in the study period

33

3 Incomplete data

9 episodes had incomplete criteria data

For PRINTO analysis

**Supplementary Figure S1.** Study flow chart. JDM= Juvenile Dermatonyositis; MCTD=Mixed Connective Tissue Disease; MRI; Magnetic Resonance Imaging; PRINTO= Pediatric Rheumatology International Trials Organisation; FU-Follow up

| Variable | Category | Summary |
| --- | --- | --- |
| Gender | Female | 16 (64%) |
|  | Male | 9 (36%) |
| Age at diagnosis | - | 7.2 ± 2.5 {3, 13} |
| Current age ^(*)^ | - | 11.2 ± 3.3 {5, 17} |
| MRI at diagnosis with feature of inflammation |  | 25 (100%) |
| JIS at presentation |  | 8-92  Mean 59  Median 60 |
| Median time between MRI scans per patient | 5 months | 5 months {2-12} IQR: 3-6 |
| The number of follow-up MRI scans for any patient |  | 1-4 |
| DMARD/Biologics used |  | Methotrexate 23  Hydroxychloroquine 16  IVIG 5  MMF 2  Rituximab 2  Cyclophosphamide 1 |
| Number of DMARD/Biologics used per patient |  | 1 in 9 patients  2 in 9 patients  3 in 5 patients  5 in 1 patient  6 in 1 patient |

*Supplementary Table S1: Patient demographics (one measurement per patient; n=25)*

Summary statistics are: number (percentage) or mean ± standard deviation {range}, (*) using age at first follow-up. MRI; Magnetic Resonance Imaging. DMARD; Disease Modifying Anti-Rheumatic Drug. IVIG; Intravenous Immunoglobulin. MMF; Mycophenolate Mofetil.

| Variable | n | Correlation Coefficient | P-value |
| --- | --- | --- | --- |
| CK | 54 | -0.05 | 0.73 |
| MMT8 | 37 | -0.15 | 0.38 |
| CMAS | 56 | -0.36 | **0.006** |
| Physician global | 54 | 0.29 | **0.03** |

*Supplementary Table S2: Associations between JIS and individual clinical measures.*

*CK; Creatinine Kinase. MMT; Manual Muscle Testing. CMAS; Childhood Myositis Activity Score.* *JIS; Juvenile dermatomyositis magnetic resonance Imaging*

*(A) (B)*

*Supplementary Figure S2: Scatterplot of (A)JIS and CMAS scores and (B) JIS and Physician Global scores*

JIS; Juvenile dermatomyositis magnetic resonance Imaging Score. *CMAS; Childhood Myositis Activity Score.*

*Supplementary Figure S3: ROC curve of JIS and clinician decision to escalate*

JIS; Juvenile dermatomyositis magnetic resonance Imaging Score.

| *Cut point* | *Sensitivity* | *Specificity* | *Correctly Classified* | *LR+* | *LR-* |
| --- | --- | --- | --- | --- | --- |
| *(>=0)* | *100.00%* | *0.00%* | *15.25%* | *1.0000* |  |
| *(>=2)* | *77.78%* | *66.00%* | *67.80%* | *2.2876* | *0.3367* |
| *(>=4)* | *77.78%* | *72.00%* | *72.80%* | *2.7778* | *0.3086* |
| *(>=8)* | *77.78%* | *78.00%* | *77.97%* | *3.5354* | *0.2849* |
| *(>=10)* | *66.67%* | *78.00%* | *76.27%* | *3.0303* | *0.4274* |
| *(>=12)* | *66.67%* | *80.00%* | *77.97%* | *3.3333* | *0.4167* |
| *(>=20)* | *66.67%* | *84.00%* | *81.36%* | *4.1667* | *0.3968* |
| *(>=24)* | *66.67%* | *86.00%* | *83.05%* | *4.7619* | *0.3876* |
| *(>=28)* | *66.67%* | *88.00%* | *84.75%* | *5.5556* | *0.3788* |
| *(>=30)* | *66.67%* | *90.00%* | *86.44%* | *6.6667* | *0.3704* |
| *(>=32)* | *66.67%* | *94.00%* | *89.83%* | *11.1111* | *0.3546* |
| *(>=36)* | *55.56%* | *96.00%* | *89.83%* | *13.8889* | *0.4630* |
| *(>=38)* | *44.44%* | *96.00%* | *88.14%* | *11.1111* | *0.5787* |
| *(>=40)* | *33.33%* | *96.00%* | *86.44%* | *8.3333* | *0.6944* |
| *(>=44)* | *22.22%* | *96.00%* | *84.75%* | *5.5556* | *0.8102* |
| *(>=62)* | *11.11%* | *96.00%* | *83.05%* | *2.7778* | *0.9259* |
| *(>=72)* | *11.11%* | *98.00%* | *84.75%* | *5.5556* | *0.9070* |
| *(>72)* | *0.00%* | *100.00%* | *84.75%* |  | *1.0000* |

*Supplementary Table S3: Detailed report of sensitivity and specificity*

| Variable | n | Correlation Coefficient | P-value |
| --- | --- | --- | --- |
| CK | 22 | -0.10 | 0.66 |
| MMT8 | 14 | -0.31 | 0.27 |
| CMAS | 23 | -0.53 | **0.01** |
| Physician global | 21 | 0.31 | 0.17 |

*Supplementary Table S4: Associations between JIS and individual clinical measures at first follow up MRI*

CK; Creatinine Kinase. MMT; Manual Muscle Testing. CMAS; Childhood Myositis Activity Score. JIS; Juvenile dermatomyositis magnetic resonance Imaging

| Variable | Category | n | JIS  Median [IQR] | P-value |
| --- | --- | --- | --- | --- |
| CID criteria | Not Met | 9 | 12 [0, 38] | 0.09 |
|  | Met | 9 | 0 [0, 4] |  |
| Clinician decision | Wean | 15 | 0 [0, 4] | **0.01** |
|  | No change | 8 | 20 [0, 47] |  |
|  | Escalate | 2 | 55 [38, 72] |  |

*Supplementary Table S5: Association between JIS and overall CID categorisation / clinician decision at first follow up MRI*

The first set of figures show the number of measurements in each category either as met or not. The second next set of figures presents the median MRI score in each category, along with a corresponding inter-quartile range. These summary measures were used due to the skewed distribution of the MRI scores. P-values indicating the significance of the association between clinical decision and JIS. CID; Clinically Inactive Disease. JIS; Juvenile dermatomyositis magnetic resonance Imaging Score.

*(A) (B)*

*Supplementary Figure S4: Boxplot of (A) JIS in patients where the CID criteria were and were not met and (B) JIS compared to clinician decision. (At first follow up MRI)*

CID; Clinically Inactive Disease. JIS; Juvenile dermatomyositis magnetic resonance Imaging Score.

| Clinician Decision | CID not met  n (%) | CID met  n (%) | P-value |
| --- | --- | --- | --- |
| Wean | 2 (22%) | 8 (89%) | 0.02 |
| No change | 6 (67%) | 1 (11%) |  |
| Escalate | 1 (11%) | 0 (0%) |  |

*Supplementary Table S6: Association between overall CID categorisation and clinician decision* at first follow up MRI

CID; Clinically Inactive Disease.

| Statistic | JIS MRI  Estimate (95% CI) | CID  Estimate (95% CI) |
| --- | --- | --- |
| Observations in analysis | 25 | 18 |
| Sensitivity | 100% (16%, 100%) | 0% (0%, 98%) |
| Specificity | 65% (43%, 84%) | 47% (23%, 72%) |
| Positive Predictive Value | 20% (17%, 56%) | 0% (0%, 34%) |
| Negative Predictive Value | 100% (78%, 100%) | 89% (52%, 100%) |
| Accuracy | 68% (46%, 85%) | 49% (34%, 64%) |
| Positive Likelihood Ratio | 2.9 (1.6, 5.0) | 0 (*) |
| Negative Likelihood Ratio | 0 (*) | 2.1 (1.3, 3.5) |

(*) Mathematically unable to calculate confidence interval.

*Supplementary Table S7: Performance of JIS and CID for the prediction of the clinician escalating treatment at first follow-up MRI*

The calculated values are shown, along with corresponding confidence intervals. CID; Clinically Inactive Disease. JIS; Juvenile dermatomyositis magnetic resonance Imaging Score.
